# Improving kidney care for people with severe mental health difficulties: A thematic analysis of personal and family members’ perspectives

**DOI:** 10.1177/13591053241254715

**Published:** 2024-06-19

**Authors:** Clodagh Cogley, Jessica Bramham, Kate Bramham, Rebekah Cheung Judge, Julie Lynch, Siobhan MacHale, John Holian, Aoife Smith, Claire Carswell, Peter Conlon, Paul D’Alton

**Affiliations:** 1 University College Dublin, Ireland; 2 King’s College London, UK; 3 St Vincent’s University Hospital, Ireland; 4 Beaumont Hospital, Ireland; 5 Irish Kidney Association, Ireland; 6 University of York, UK; 7 Queen’s University Belfast, UK

**Keywords:** adherence, bipolar, depression, dialysis, healthcare access, kidney disease, mental health, mental illness, psychosis, schizophrenia

## Abstract

People with severe mental health difficulties (SMHDs) often have poorer access to kidney healthcare. To better understand the barriers and facilitators to kidney healthcare for this population, we conducted interviews with nine individuals with SMHDs and four family members. Through reflexive thematic analysis, we generated three themes: (1) ‘*One size doesn’t fit all*’ describes the need for individualised kidney healthcare, adapted to meet the specific needs of each person with a SMHD. (2) ‘*You just can’t say, “I’m only dealing with your kidney here”*’ describes how fragmentation of physical and mental healthcare services can lead to poorer outcomes for people with SMHDs, underscoring the need for coordinated care. (3) ‘*Just treat me with respect*’ describes the impact of healthcare provider attitudes. Overall, participants praised the dedication and kindness of renal clinicians. However, some participants also described experiences of stigma and discrimination, and called for additional education for healthcare providers regarding SMHDs.

## Introduction

People with severe mental health difficulties (SMHDs), including psychosis, schizophrenia, bipolar disorder and major depression, die 10–20 years earlier than those without SMHDs (Hjorthøj et al., 2017). The majority of these premature deaths are caused by chronic health conditions, including cardiovascular, metabolic and kidney diseases. Research indicates that inequitable access to quality healthcare for people with SMHDs plays a significant role in this disparity (O’Connor et al., 2023).

Chronic kidney disease (CKD) occurs when the kidneys can no longer adequately filter blood, leading to the build-up of excess fluid and waste in the body. This can lead to other health problems, including stroke and cardiovascular disease (Levey and Coresh, 2012). When kidney failure occurs, renal replacement therapy (dialysis or transplant) is needed for survival. People with SMHDs have a higher risk of developing kidney disease, due to a range of factors, including the use of lithium, as well as higher rates of cardiovascular disease, type 2 diabetes and smoking (Carswell et al., 2023). Research using structured clinical diagnostic interview indicates that the prevalence of Major Depressive Disorder is 19% in people with CKD (Watnick et al., 2005). An estimated 3% of people with kidney disease have received a diagnosis of schizophrenia, schizoaffective disorder, psychosis or bipolar disorder (Cogley et al., 2023a).

For people with kidney disease, those with SMHDs have higher mortality rates and receive suboptimal kidney healthcare (Carswell et al., 2023; Kimmel et al., 2019). For example, individuals with kidney failure and SMHDs have higher rates of hospitalisations, particularly through the emergency department (Kimmel et al., 2019; McPherson et al., 2014). They also have fewer appointments with renal clinicians (Hsu et al., 2015). Moreover, people with SMHDs are less likely to receive a kidney transplant compared to those without SMHDs (Bitan et al., 2019; Iwagami et al., 2018), notwithstanding evidence that, following careful assessment for suitability, people with SMHDs have comparable kidney transplant outcomes to those without SMHDs (Butler et al., 2017; Kofman et al., 2018).

Despite the known disparities in health outcomes and access to kidney healthcare for people in this population, research investigating kidney healthcare for people with SMHDs is limited. To our knowledge, the only existing studies are from the perspective of HCPs (Alwar and Addis, 2022; Cogley et al., 2023b). These studies indicate that many people with kidney disease and concurrent SMHDs face additional challenges accessing kidney healthcare due to fluctuations in mental state, depression, cognitive difficulties and mistrust of healthcare professionals (Cogley et al., 2023b). HCPs also described how stigma towards people with SMHDs and lack of mental health training for renal clinicians contribute to poorer outcomes for this population (Alwar and Addis, 2022; Cogley et al., 2023b). Compared to other health conditions, HCPs’ accounts suggest that kidney healthcare poses additional and unique challenges for people with SMHDs, due to the high burden associated with treatment (Cogley et al., 2023b).

To our knowledge, there is no existing research on kidney healthcare for people with SMHDs from the perspectives of the individuals themselves, nor from their family members. Given the far-reaching impact that kidney disease and SMHDs have on individuals and their families, HCPs may not be aware of many of the barriers and facilitators to kidney healthcare that they experience. Thus, research including their perspectives is essential to ensure any future interventions are relevant, meaningful and responsive to their needs, in line with the ideals of person-centred care. To better understand how to improve kidney healthcare for people with SMHDs, we conducted semi-structured interviews with (a) people with kidney disease and concurrent SMHDs and (b) their family members. We aimed to address two research questions:

1. What are the barriers and facilitators to effective kidney healthcare for people with SMHDs?
2. How might kidney healthcare for people with SMHDs be improved?

## Methods

This study was conducted as part of a larger research project investigating kidney healthcare for people with SMHDs (Carswell et al., 2023; Cogley et al., 2022, 2023a, 2023b). The research design and implementation was guided by a PPI advisory group, consisting of three individuals with kidney disease and SMHDs. This study received approval from University College Dublin Research Ethics Committee (HS-21-19-D’Alton-Cogley).

### Participants

The study enrolled 13 participants, including nine people with kidney disease and concurrent SMHDs, and four family members. For participants with kidney disease and SMHDs, the inclusion criteria were: (a) have a diagnosis of CKD stages 3–5; (b) have a concurrent diagnosis of Major Depressive Disorder, Bipolar Disorder or a psychotic disorder; (c) have received kidney healthcare in Ireland; and (d) have capacity to give informed consent, confirmed through a comprehension assessment, conducted by CC. Family participant had to be: (a) a relative of a person with stage 3–5 CKD with a SMHD who had received kidney healthcare in Ireland and (b) able to provide informed consent. Two SMHD participants had participating family members, others were not related. All participants were fluent in English. For participant demographics see Table 1. For more information regarding the system of kidney healthcare in Ireland, see Cogley et al. (2023b).

**Table 1.** Summary of participant demographics at time of interview.

**Demographic information of people with CKD and concurrent SMHDs (*n* = 9)**	
Age	Mean: 46 years; Range: 24–69 years; SD = 14.21
Gender	Male: 5 Female: 4
Mental health diagnosis	Major depressive disorder: 2
	Bipolar disorder: 3
	Schizoaffective disorder: 1
	Schizophrenia: 1
	Psychosis: 1
	Major depressive disorder and borderline personality disorder: 1
Current RRT	Transplant: 5 Haemodialysis: 3 None: 1 (Stage 4 CKD)
Time since developing CKD	Mean 21.22 years; Range 5–36 years; SD = 12.1
Ethnicity	White: 7
	Non-white: 2
Living situation	At home with partner: 4 At home with housemates: 1 At home alone: 1 Supported accommodation in mental health setting: 3
Employment status	Retired: 3 Sick leave: 2 Unemployed: 3 Student: 1
**Family demographic information (*n* = 4)**	
Age	Mean: 61; Range: 41–73; SD = 8.4
Gender	Male: 1 Female: 3
Ethnicity	White: 4
Relation to patient	Wife: 1
	Husband: 1
	Sibling: 2

CKD: chronic kidney disease; RRT: renal replacement modality; SMHD: severe mental health difficulty.

The study was advertised on the Irish Kidney Association Twitter page, and Nephrology and Psychiatry clinicians facilitated recruitment two Dublin-based hospitals. Treating clinicians approached potential participants for interview. It was emphasised that participation was completely voluntary and anonymous, and that declining would not impact their care. Two individuals with SMHDs declined to participate and two others were unable to provide informed consent, as indicated by the comprehension assessment. All approached family members participated.

## Procedure

The interview schedule was informed by existing literature (Happell et al., 2012; Ladin et al., 2009), with input from the PPI group (see Appendix A for interview schedule). Semi-structured interviews were conducted with a researcher (CC) in person (*n* = 6), or via phone (*n* = 3) or via videoconferencing platform (*n* = 4). There was no monetary compensation for participation. Interviews took place between June 2021 and April 2023, and lasted 32–121 minutes (*M* = 57).

### Analysis

We conducted a reflexive thematic analysis of interview data, within a largely critical realist framework (Braun and Clarke, 2019). See Table 2 for details.

**Table 2.** Process of thematic analysis. Braun and Clarke describe their approach to thematic analysis as ‘reflexive’, as it emphasises the active role of the researcher in the interpretation of data and generation of themes (Terry et al., 2017). Their gauges of quality include researcher reflexivity, systematic and rigorous coding and theoretical knowingness (Braun and Clarke, 2019).

**Phase**	**Process and author involvement**
**Phase 1: Data familiarisation**	CC conducted interviews, noting insights and impressions. Interviews were transcribed verbatim. CC removed identifying information, assigning pseudonyms. CC read the data repeatedly to facilitate deep familiarisation, discussing impressions with her academic supervisors, JB and PD. Throughout data collection and analysis, CC used a reflexive journal to critically examine her own assumptions, biases and positionality.
**Phase 2: Coding**	CC, RCJ and JL systematically and independently conducted open coding. Participants’ accounts were interpreted as ‘real’ while recognising that they are influenced by social structures, power relations and other contextual factors. The use of thematic analysis was largely inductive and descriptive, using open coding and emphasising data-based meaning. Codes were organised using NVivo 12 software. CC, RCJ and JL wrote notes regarding their initial impressions of the data. To foster reflexivity and enhance understanding of the data, CC then engaged in discussions with RCJ and JL regarding the findings.
**Phase 3: Generating initial themes**	CC generated the initial themes, relating to barriers and facilitators to kidney healthcare for people with SMHDs, by clustering together related codes based on patterns of shared meaning.
**Phase 4: Reviewing and refining themes**	CC, RCJ and JL reviewed the initial themes. Through discussion, themes were reworked until they were considered as providing a ‘good fit’ with the data. CC then reviewed themes against the coded data and the full dataset.
**Phase 5: Defining and naming themes**	CC refined the ‘story’ of each theme, selected illustrative quotations, finalised theme names and wrote the results section. This was reviewed by all authors.
**Phase 6: Producing the report**	CC wrote and completed the final paper, with input from all other authors.

CC is a PhD researcher with a background working in health psychology, who has experience working clinically with people with SMHDs and chronic health conditions. CC does not have kidney disease or a SMHD, and did not know participants prior to interviews. RCJ is a Renal Research Fellow and JL is a Clinical Psychologist in Nephrology.

## Results

As the general challenges associated with kidney disease and its treatment have been well-documented elsewhere, our analysis focuses on the barriers and facilitators to care that are specific to people with severe mental health difficulties (PwSMHDs). Three themes were generated: ‘One size doesn’t fit all’, ‘You just can’t say, “I’m only dealing with your kidney here” ’ and ‘Just treat me with respect’ (see Table 3).

**Table 3.** Summary of barriers and facilitators to effective kidney healthcare for people with severe mental health difficulties (pwSMHDs).

**1.*‘One size doesn’t fit all’***	
**Barriers**: • Lack of flexibility in the provision of healthcare for people with SMHDs, to accommodate for: • Anxiety • Depression • Mood fluctuations • Suicidality • Cognitive difficulties • Difficulty following treatment requirements • Lack of government-provided supports in the community • Unsuitable hospital ward settings • Limited social support • Financial difficulties	**Facilitators**: • Flexible, individualised care reflecting the needs and preferences of each PwSMHD • Assessment of mental health, close monitoring and immediate intervention where needed • Regular screening for suicide risk • Availability of psychiatry and psychological support • HCPs taking time to adequately address concerns • Predictable routines • Involvement of family • Adjusting delivery of information based on capacity • Additional support for following treatment requirements, diet and appointment attendance • Prompting and transport for appointments
**2. *‘You just can’t say, “I’m only dealing with your kidney here” ’***	
**Barriers**: • Fragmentation and separation of physical and mental healthcare services • Renal services not attending to mental health • Mental health services not addressing physical health needs (i.e. health monitoring, diet, appointment attendance, etc.) • Underfunding and understaffing of mental health services • Bureaucracy limiting family involvement • Renal HCPs’ lack of understanding about SMHDs	**Facilitators**: • Coordinated, ‘whole person’ approach to person’s overall care • Communication and collaboration between physical and mental healthcare services • Care coordinator or key worker taking responsibility for ‘big picture’ of person’s care • Education from MH service workers on kidney disease • Mental health services providing dialysis-adherent meals • Renal HCPs attending to mental health needs • Psychiatry and psychology input on renal MDTs • Careful multidisciplinary management of psychiatric medications
**3. *‘Just treat me with respect’***	
**Barriers**: • Stigma and discrimination towards PwSMHDs • Concerns of PwSMHDs being dismissed by HCPs • PwSMHDs being ‘blamed’ for difficulty following treatments • Impatience regarding the additional needs of PwSMHDs in healthcare settings	**Facilitators**: • Kind, dedicated and compassionate HCPs • Treating PwSMHDs with respect and humanity • Long term, trusting relationships with HCPs • HCPs’ patience, sensitivity and professionalism • Education for HCPs regarding SMHDs

### ‘One size doesn’t fit all’

People with SMHDs and their family members described the many ways in which SMHDs impact their lives and access to kidney healthcare, and highlighted the need for care that is adapted to the specific needs of each person with a SMHD: ‘We have to wake up and start realising that doctors cannot treat everyone exactly the same. One size doesn’t fit all’ (Linda, family member). For example, some participants described experiencing high levels of anxiety, which can exacerbate their health concerns to a ‘totally consuming’ level (Liam, PwSMHD). Some participants noted that anxiety makes them more sensitive to physical symptoms and, in some cases, can cause other physical problems:

I couldn’t get through to any of the medical team, no matter how much I tried. And that led to severe anxiety in me, and then that severe anxiety starts these other issues, like eating, sleeping, energy…and then heart and bowel and all sorts of things start going. (Robert, PwSMHD)

These participants described how passing comments from HCPs regarding their health or treatment could send them ‘into a panic’ (Mary, PwSMHD). They emphasised the need for additional sensitivity from healthcare professionals, as well as extra time so that their concerns can be adequately addressed. Being informed about what to expect and having a predictable routine were also described as helpful.

For some participants, the hospital setting caused anxiety: ‘The dialysis unit was so busy and loud, like a gambling casino. It’s really overpowering and traumatic when you’re not used to it’ (David, PwSMHD). One participant who experienced psychosis following her kidney transplant described how the noises on the ward triggered distressing auditory hallucinations. She described how simple accommodations, such as nurses taking time to sit with her or being moved to a quiet room, significantly decreased her distress:

There were so many triggers in hospital. (…) I started to hear things, that I was being talked about, that I’d done something terrible, that I brought Covid into the hospital and I’d killed loads of kidneys. And the difficulty became, I couldn’t tell the difference between what I really heard and what I thought I heard. So when they gave me the room with windows it did help lift my spirits, because I could look out and see and start thinking I was going to be back out there. And the nurses were so kind, they would sit with me. (Jennifer, PwSMHD)

The same participant described how being given medication to manage her symptoms of psychosis was not sufficient. She emphasised that she needed to speak with mental health professionals, who helped make sense of her experiences and gave her practical coping strategies for distressing hallucinations: ‘I needed someone to talk to me about what I was going through’ (Jennifer, PwSMHD).

Participants described how during periods of depression, they can struggle with motivation to follow treatment requirements, follow up with doctors and advocate for their own care: ‘My brain just shuts down completely. So I have no energy or will do to anything’ (Robert, PwSMHD). For some, the effects of depression are so debilitating that even basic self-care is challenging:

When I’m depressed I’m just really down, and numb. It isn’t fun. It’s hard to even get out, get up. It’s hard to even go to the toilet when you’re numb, or go down the stairs. It’s hard on your body because you feel so like…not worth anything. So it’s very difficult to just get on with something. (Nancy, PwSMHD)

Many participants reported that they require additional support to attend medical appointments, prepare dialysis-adherent meals and advocate for their care. However, there are few government supports available, and financial constraints further limit participants’ access to appropriate supports. As a result, the responsibility often falls on the family, which can be a source of significant strain: ‘As family carer … you’re dragged to your knees. And really, the health services would be quite happy for you to go on until you die. That’s not an exaggeration’ (Sarah, family member). Some participants with SMHDs described having little social support:

I don’t have family here. So when it comes to all my medical and my mental health appointments, I have to deal with everything on my own. I don’t have anybody there that can support me and can take on board what’s happening. (Rachel, PwSMHD)

Participants described how cognitive difficulties, mood swings and low motivation can impact the suitability of certain treatment options. For example, participants described how the time and effort needed to administer peritoneal dialysis at home was ‘overwhelming’ (Nancy, PwSMHD) when they were in a state of depression. These participants were subsequently placed on haemodialysis, which required less organisation and motivation on their behalf.

The issue of suicide risk was also raised. Two participants with SMHDs reported that they had previously attempted suicide by ceasing their home-based renal replacement treatment, or by eating foods that are potentially dangerous for people on dialysis. Another participant reported that he was in a state of depression when he received the call for his kidney transplant, and would have declined the transplant if his wife had not been there. Participants highlighted the need for ongoing assessment for suicide risk in people with SMHDs and kidney failure. The importance of psychiatry and psychology input was also discussed, to facilitate immediate interventions where needed: ‘Counselling has saved my life, there’s no doubt about it’ (Liam, PwSMHD). Participants also reported that psychological support helped to improve their ability to follow treatment requirements.

Most participants with SMHDs described having a good understanding of kidney disease and the associated treatments. However, cognitive difficulties can impact the ability of some people with some people with SMHDs to understand medical jargon and remember treatment requirements: ‘He just won’t understand you telling him that his potassium is down, it may as well be in Dutch’ (Sarah, family member). Participants described how HCPs need to adjust their communication style according to the needs of each person. For example, some individuals with SMHDs need information to be broken down into smaller pieces and explained in simpler language. Participants also recommended providing simple and direct written instructions. For those with cognitive difficulties, participants emphasised the need to involve carers or family at appointments, to help understand and retain information. Participants also described needing additional prompting for appointments and support with transport: ‘I shouldn’t have to do that, they should be reminding him and booking him a taxi’ (Sarah, family member)

### ‘You just can’t say, “I’m only dealing with your kidney here”’

Participants reported that the fragmentation and separation of physical and mental healthcare services are significant barriers to kidney healthcare for people with SMHDs. Participants were frustrated that renal services often don’t attend to mental health needs, and that mental health services don’t address physical health needs:

For people who have several physical and mental health comorbidities, you just can’t say, “I’m only dealing with your kidney here”. Because the whole person is involved. You can’t take your kidney out and leave it on the table. (Mary, PwSMHD)

The issue is this silo mentality, particularly in psychiatry. There’s this mentality of ‘Well, I’m only looking after the right ear and you can look after the left … ’ (Linda, family member)

Participants emphasised the need for a coordinated approach to the person’s overall care. However, while some participants reported that their renal and mental health services are in close communication, others reported little collaboration between services. These participants felt that no one takes accountability for the person’s overall health and wellbeing, or acknowledges the interaction between kidney disease and SMHDs. For example, one family member described frustration about neither the hospital nor the person’s mental health services taking responsibility for managing their medical appointments, or their medication and diet requirements:

I don’t know what game the mental health services are playing at, but not one of them has ever asked me about his kidney disease. They don’t give a flying fig. So they don’t monitor. They’ll let him drink coffee all day, which he shouldn’t, because his kidneys are failing. (Sarah, family member)

Similarly, mental health services do not routinely monitor the physical health of people with SMHDs, nor adequately inform them of the potential health risks associated with psychiatric medications: ‘Nobody warned me, and now none of them are taking responsibility for what they’ve done to my kidneys’ (Rachel, PwSMHD). Participants reported that mental health services are chronically underfunded and understaffed, which limits their ability to meet the health needs of people with SMHDs. As a result, participants described having to advocate for basic services: ‘They just make it so, so difficult. I shouldn’t have to get a politician’s support. I shouldn’t have to write five page letters to them to have my basic needs met’ (Rachel, PwSMHD).

Participants described the need for a care coordinator or key worker, who could help link the person’s physical and mental health care, advocate on their behalf and assist with appointments:

Every single day I’m taking phone calls. Whereas I would argue that if they had a support person, that they should be doing this work. Because a lot of it is pure grunge work, like ringing for a taxi, ringing the GP, checking the blood tests have gone in. It would be so lovely for me to think that if I couldn’t go to an appointment, someone else would be there. And then I wouldn’t feel this pressure all the time to be there to take in what was said. (Linda, family member)

Some participants identified the lack of supports provided in assisted accommodation as a barrier to effective kidney healthcare. For example, one family member described how her sibling was in ‘low support accommodation’ which has few supports for tenants with SMHDs but also limits her ability to assist in her sibling’s care:

It makes no sense, but the “supported housing” isn’t supported. So I have to do everything, but I actually have no authority to do anything. So I have to ask their permission for everything, even to hang up a nail. Even though they aren’t prepared to help. (Sarah, family member)

This family member reported that if her sibling was perceived as having less capacity, he would get better supports: ‘The nurse manager said “Well, the real problem is if his mental health issues were more severe, we’d be able to do more for him.” Now, how is that logical?’ In contrast, a participant described how his accommodation with higher levels of support provides dialysis-adherent meals, and ensures he gets adequate exercise each day. Two family members felt that mental healthcare services often ‘hide behind’ (Linda, family member) bureaucracy and data protection legislation, to avoid having to deal with family members who are advocating for better care on behalf of people with SMHDs: ‘It’s much easier for psychiatric services if the families aren’t involved, to hold them accountable. Because otherwise, I mean, they can just get away with not providing these services. I think it’s a scandal, myself’ (Sarah, family member).

Participants appreciated when renal HCPs address their SMHDs like any other aspect of their care. However, some described how they the topic of mental health is rarely discussed: ‘I’m fairly sure they never even think about mental health, let alone ask about it’ (Liam, PwSMHD). Some participants described how this can add to the perception that SMHDs are a taboo subject: ‘It implies there’s a stigma. It almost implies that I did something wrong’ (Liam, PwSMHD). While some renal clinicians were described as having a good understanding of SMHDs, others need further education regarding how mental health can impact kidney healthcare:

I’m not expecting them to be experts. But I think if the doctors had a basic understanding, it would be easier to have a conversation about mental health with them. Because I can’t have a conversation with my doctor about my mental health, I don’t. Because there’s no point. (Nancy, PwSMHD)

Participants stressed the importance of having mental health professionals in renal settings, including psychiatrists, psychologists and social workers. Mental healthcare professionals often educated the renal team, informed the person’s kidney healthcare, and advocated on their behalf. For example, one participant with a SMHD (Jennifer) described how a liaison psychiatrist helped her medical team understand why she was experiencing psychosis post-transplant, and developed a protocol so they could respond appropriately. Participants also described the need for psychological support provided in the hospital:

I think the day I was told I had kidney failure, I should have been sent to a counsellor. (…) It was like a pressure cooker, and then the lid blew, and then I tried to kill myself. It may not have come to that had I had some relief from speaking about it, you know? (Liam, PwSMHD).

Participants also stressed the importance of close collaboration and communication between renal and psychiatric services in the management of psychiatric medications. In particular, participants described the careful balancing act of considering the impact of lithium on the kidneys against the need to manage their SMHD:

The medications for the kidney and for the bipolar are in conflict. So it took years to figure out how much they could give of each. It was kind of hit and miss for a long time. And even now, they’re saying the lithium, it’s still having some sort of an effect on the kidney. But they’ve tried and tested different levels, and they’ve come to a decision that the amount that she’s on now is acceptable for the kidneys. (Ethan, family member)

Participants stressed the need to carefully consider the risks of changing medications, closely monitor the person’s progress and respond immediately to any deteriorations in their mental health. In cases where changes in psychiatric medications were not appropriately managed, participants described extreme adverse reactions which had long term impacts on their health and wellbeing:

I had a very bad breakdown, I was suicidal. And I ended up six months in hospital, and another year in day care. Because they adopted a “wait and see” attitude. And eventually then they put me on the Seroquel. So to me that was very poor. Very poor, they should have put me on an equivalent drug to manage it, rather than wait ‘til I had the crash and then give it to me. (Liam, PwSMHD)

Participants also emphasised the importance of considering their preferences, as some people with SMHDs advocated to stay on or return to lithium, despite its potential impact on the kidneys:

I think this is true of many persons who’ve been to psychiatric facilities, it is that mental health is more important than the physical health. I would much rather leave this room with pancreatic cancer than leave it with even a suggestion of the old terrors. (David, PwSMHD)

Participants reported that small efforts to coordinate the physical and mental health of people with SMHDs could improve outcomes and ultimately save unnecessary health services expenditure on hospitalisations: ‘Just a phone call, a short email. I’m not asking everyone to hold hands for a month. It’s just link. Keep him well. Keep him out of hospital’ (Linda, family member).

### ‘Just treat me with respect’

Participants described the significant impact that clinician attitudes have on their kidney healthcare, health outcomes and overall wellbeing. Overall, participants praised their renal HCPs, describing them as kind, dedicated and compassionate:

Everyone, every nurse, every doctor in this hospital I’ve seen, I’ve talked to, is so great at their job. It’s great, because when you’re down and sick, you need people that you can talk to, and who you can have a laugh with. (Nancy, PwSMHD)

Most of all, participants appreciated when HCPs treat them with humanity, empathy and respect. Participants described the positive impact that even one HCP can have on their care: ‘I’m just very lucky that there’s a nurse there who is so committed, so dedicated. I don’t know what we would do without her’ (Sarah, family member). Similarly, participants described the benefits of having long-term relationships with renal staff, who understand the nature of their SMHD and how it impacts their care.

However, a number of participants described instances whereby renal and mental health services showed little dedication towards people with SMHDs: ‘And I actually said, “It’d be more convenient for you if (family member’s name) died, and he’d be another one off the books.” And it’s the truth’ (Sarah, family member). Participants reported that some healthcare professionals don’t take the time to speak to them or understand their experiences. Instead, the focus seems to be centred solely on prescribing medications: ‘They just treat me like a another number on the chart. All they say is “Just give him some tablets there”’ (Ryan, PwSMHD).

While some participants reported never having experienced stigma, others described multiple times whereby HCPs had discriminated against them on the basis of their SMHD. For example, participants described how HCPs made assumptions about them based on their SMHD diagnosis:

As soon as they read the list of scripts, I could see that it predisposed them toward almost a Munchausen sense of the patient in the bed…as needy and difficult and demanding and contrary. (David, PwSMHD)

Some participants described instances in which their concerns were dismissed because of their SMHD: ‘They’re not listening to people with mental health difficulties. In other words, they say, “Ah, yeah, he’s got mental health difficulties, just ignore him”’ (Liam, PwSMHD). Many participants described having to constantly advocate for and explain themselves, as some HCPs underestimate the impact of SMHDs on their capacity to engage with treatment:

I have a bad memory and my doctor doesn’t seem to think I have a bad memory. Even when I’m trying to explain to him, he thinks that I just don’t want to take them. And I’m trying to tell him, I forget, even if I have an alarm set. But he thinks that I just don’t want to take them. (sighs) It’s frustrating. (Nancy, PwSMHD)

Similarly, participants described how some HCPs resent having to spend additional time with them, and respond impatiently to their requests:

He said “I can’t spend all this time on you, you’re not the only person here.” But what they don’t understand is my hyper anxiety, I have a sensitized system. So anxiety to me is, compared to you, it’s completely different. I’m not resilient in the same way. So I need that extra time to have things explained to me. It’s not my fault. (Robert, PwSMHD)

Participants described how these negative interactions impact their ability to trust HCPs and engage in care. Some participants understood that, in the context of an under-resourced healthcare system, providing care to people with SMHDs can sometimes present challenges for HCPs. However, these participants felt they should still be able to respond with understanding and professionalism:

So when I’m in a high or manic state, I can lose the sense of myself a bit. There’s no calm or balance in my conversation. Even with dealing with consultants. I can be pretty vocal and might say very direct stuff that normally I wouldn’t be saying, like “you’re not dealing with this properly”. So in some cases you can drive them to the limit, and I suppose I understand that. But if they know it’s because I have a mental health difficulty they should be able to respond a bit more patiently. Other professionals have been far more understanding. They didn’t get wrapped up in what I was saying, or went on to something else, or dealt with it in a more professional way. (Robert, PwSMHD)

Some participants felt that stigma and discriminations stems from HCPs’ lack of understanding about SMHDs, and suggested additional education for HCPs. One participant advocated for increased involvement of peer support workers with lived experience of SMHDs, who would be able to advocate on their behalf: ‘There is a big improvement with peer support workers becoming involved. But they’re not included in the team network as much as they should be’ (Rachel, PwSMHD). Participants also emphasised the need for society-wide change in how people with SMHDs are viewed: ‘We have a long way to go in this country, in terms of how we treat people with mental health difficulties’ (Sarah, family member).

## Discussion

To our knowledge, this is the first study to examine kidney healthcare from the perspectives of people with SMHDs or their family members. Findings provide numerous insights into how to improve kidney healthcare for this population. The results demonstrate how SMHDs can influence access to care in various ways, underscoring the importance of individualised kidney healthcare which is adapted to meet the specific needs of each person. Participants’ accounts highlight the risk of suicide in individuals with kidney disease and concurrent SMHDs, and the importance of close monitoring and immediate intervention when needed. Participants also described how the fragmentation and separation of physical and mental healthcare services can result in poorer outcomes for people with SMHDs, emphasising the need for a coordinated approach to care. Results indicate that renal teams should be adequately resourced with psychiatry and psychology professionals, to inform the care of people with SMHDs and advocate on their behalf when needed. Participants also described the significant impact of HCP attitudes. While participants praised their renal clinicians’ dedication and kindness, some also described experiences of stigma and discrimination on the basis of their SMHD. Participants emphasised the need for additional education for HCPs regarding mental health, as well as society-wide change in how people with SMHDs are treated.

Participants’ accounts were largely consistent with research regarding access to general healthcare for people with SMHDs (Druss, 2007; Happell et al., 2012). However, a number of barriers specific to kidney healthcare for this population were also discussed. For example, some people with SMHDs have additional difficulty following complex renal replacement treatment and diet requirements, due to depression, cognitive difficulties, lack of social support and financial constraints (Cogley et al., 2023b). Findings illustrate that a large network of comprehensive and coordinated support is needed to help individuals with SMHDs face the range of daily challenges associated with kidney disease. However, due to inadequate government-provided supports, the responsibility often falls on family members.

There are a number of existing positive supports for people with kidney disease and their family members who are experiencing mild to moderate psychological distress, including self-management programmes, and counselling and peer support provided by the Irish Kidney Association. However, our findings indicate that individuals with SMHDs require additional assistance which reflects the level of functioning of each person, to ensure they receive the help they need and want to stay well, while also respecting their rights to autonomy.

Our findings also highlight the risk of suicide in people with kidney disease and concurrent SMHDs. Given the high rates of suicide attempts in people with kidney disease with mental health difficulties (Cogley et al., 2023a, 2023b), findings underscore the need for regular screening for suicide risk in this population. As multiple participants described attempting suicide by ceasing their renal replacement therapy, passive suicidal ideation should be thoroughly investigated. The availability of appropriate mental health supports is also vital, to ensure individuals have access to collaborative assessment and management of suicidality, psychological therapy, safety planning and pharmacotherapy. Our findings add to existing evidence that Nephrology departments should have comprehensive multidisciplinary team-based care including psychiatry, psychology, clinical nurse specialists and social work (Cogley et al., 2023a, 2023b). Research indicates that most Nephrology services lack such supports (Seekles et al., 2019).

It is well documented that the separation between mental and physical healthcare leads to poorer outcomes for people with SMHDs (Druss, 2007; Happell et al., 2012; O’Connor et al., 2023). Increased communication and coordination between renal and mental health services can help to inform nephrology professionals of the specific needs of each person with a SMHD, and maintain continuity of care (Cogley et al., 2023b). Increased communication between services also has the potential to improve renal care knowledge for mental health professionals, which may improve their ability to support engagement in healthcare assess suicide risk for this population (Cogley et al., 2023b).

Participants’ accounts indicate that a care coordinator could help people with kidney disease and concurrent SMHDs to access kidney healthcare in Ireland. Such roles have been created in other countries to help individuals with SMHDs, by supporting self-management goals, monitoring and following-up with care, assisting with appointments, communicating knowledge, developing care plans and aligning resources with the person’s needs (McDonald et al., 2014). Further research is needed to determine the acceptability, feasibility and utility of involving care coordinators in kidney healthcare of individuals with SMHDs in Ireland.

Findings add to evidence that people with SMHDs are often treated with a lack of dignity and respect by healthcare professionals, and are more likely to have their health-related concerns dismissed or questioned for credibility (Happell et al., 2012; O’Day et al., 2005). When SMHDs affect HCPs’ evaluation of physical health symptoms, this is known as ‘diagnostic overshadowing’ (Jones et al., 2008). This can result in under-recognition of physical health issues, and inadequate or delayed medical care (Molloy et al., 2023). Research suggests that the most effective stigma-reducing interventions for HCPs include testimonies from individuals with lived experience of SMHDs, and target unconscious biases that may negatively impact care (Knaak et al., 2014). HCPs should also recognise that individuals with SMHDs also need additional consultation time, to adequately address their concerns (Happell et al., 2012).

### Strengths, limitations and directions for further research

Our study included individuals with a range of SMHDs and associated difficulties, contributing to a more comprehensive understanding of the barriers and facilitators to care for this population. This is a notable strength, as research often fails to include the perspectives of individuals with SMHDs. Interviewing family members gave us insight into a number of the barriers and facilitators to kidney healthcare faced by people with SMHDs who were unable to provide informed consent. However, the sample size was small, and research with a wider range of people with SMHDs and their family members is needed to determine whether findings are transferrable to other settings.

Furthermore, participants were English speaking and primarily White. As participants were recruited through hospitals and the Irish Kidney Association, the views of individuals not engaged with these services were not represented. Additionally, as clinicians determined who to approach, individuals with less positive relationships with their clinicians may have been less likely to participate. Research including a more diverse sample of individuals with SMHDs and family members may capture additional barriers and facilitators to effective kidney healthcare experienced by this population.

Future research should prioritise interventions which provide comprehensive, person-centred support for people with kidney disease and concurrent SMHDs. Examples include investigating the utility of care coordinators, and ways to support following treatment requirements.

## Conclusions and clinical implications

The perspectives of people with SMHDs and their family members provide valuable insights into how to improve kidney healthcare for this underserved population. As SMHDs can influence engagement with care in various ways, kidney healthcare should be individualised to meet the specific needs of each person with a SMHD. For example, some individuals with SMHDs may need additional assistance to follow complex treatment and diet requirements. Participants’ accounts also highlight the risk of suicide in individuals with kidney disease and concurrent SMHDs, and the importance of screening for suicide risk in this population. Findings add to evidence that renal teams should be adequately resourced with psychiatry and psychology professionals, to inform the care of people with SMHDs, and facilitate appropriate interventions when needed. This study also demonstrates how the separation of physical and mental healthcare services can result in poorer outcomes for people with SMHDs, emphasising the need for a coordinated approach to care. Findings suggest that having an assigned care coordinator could help individuals with SMHDs access kidney healthcare, although further research is required to determine the utility and feasibility of such a role. Participants described how renal clinicians’ dedication and kindness positively impacts their kidney healthcare and overall wellbeing. However, experiences of mental health-related stigma and discrimination were also described. Our results indicate the need for additional education for HCPs regarding mental health, as well as society-wide change in how people with SMHDs are treated.

## Supplemental Material

sj-docx-1-hpq-10.1177_13591053241254715 – Supplemental material for Improving kidney care for people with severe mental health difficulties: A thematic analysis of personal and family members’ perspectives Supplemental material, sj-docx-1-hpq-10.1177_13591053241254715 for Improving kidney care for people with severe mental health difficulties: A thematic analysis of personal and family members’ perspectives by Clodagh Cogley, Jessica Bramham, Kate Bramham, Rebekah Cheung Judge, Julie Lynch, Siobhan MacHale, John Holian, Aoife Smith, Claire Carswell, Peter Conlon and Paul D’Alton in Journal of Health Psychology
