# Supplementary material for: Improving kidney care for people with severe mental health difficulties: A thematic analysis of personal and family members’ perspectives
Source: J Health Psychol. 2024 Jun 19;30(5):1044–58. doi: 10.1177/13591053241254715 (PMC11977831; doi:10.1177/13591053241254715)
Supplement: sj-docx-1-hpq-10.1177_13591053241254715 – Supplemental material for Improving kidney care for people with severe mental health difficulties: A thematic analysis of personal and family members’ perspectives [file sj-docx-1-hpq-10.1177_13591053241254715.docx]

Appendix A. Semi-structured interview schedules

**Introduction**

I am interested in your experiences and your beliefs about the kidney disease care for patients with MHD. Sharing your story and your insights will help us to understand how care for individuals with mental health difficulties could be improved.

This interview should last about one hour in total. I will audio record our conversation on an electronic recording device. After I have transcribed (written out) the interview and removed any identifying information, the audio recording will be deleted. I’m interested in hearing all of your ideas - there is no right or wrong answer. Just respond as honestly as you can. As a reminder your participation is very much appreciated, however it is completely voluntary. If you would like to opt out at any stage that is quite ok. If you don’t want to answer a particular question that is also ok, just ask me to move onto the next question. Also, if you would like to take a break at any time just let me know and we can pause the interview.

If you have any questions before the interview begins, or during the interview, please let me know and I would be happy to answer them.

**Patient interview schedule:**

**Background**

Could you tell me when you were diagnosed with kidney disease?

Have you been on dialysis?

Have you received a kidney transplant? When?

Do you have any other medical conditions?

Age?

Gender?

Employment status?

Living situation?

Relationship status?

Ethnicity?

Can you please begin by telling me what type of mental health difficulty you have?

When diagnosed?

Have you been hospitalized?

Currently taking medication?

**Access to care**

Have you ever had difficulty accessing quality healthcare for your kidney disease?

Has your mental health difficulty made any aspect of kidney care more difficult?

*ESKD Diagnosis:*

Can you tell me a little bit about your diagnosis of kidney disease? When, by who, etc

What was the most difficult part of receiving your diagnosis?

Do you think your mental health difficulty made any aspect of diagnosis more difficult?

What helped care go well at this stage?

How could care be improved at this stage?

*For patients who have been/were on dialysis:*

How do/did you find dialysis? What is the most difficult aspect of attending dialysis for you?

Did you feel your mental health difficulty impacts dialysis?

In your experience, what helps dialysis go well?

What supports would be most helpful to you, to help dialysis go well?

How do/did you find the dietary and fluid restrictions? What is the most difficult aspect for you?

What makes sticking to diet and fluid restrictions easier? What supports would help you stick to diet and fluid restrictions?

*Transplant:*

Could you tell me a little bit about your experience of receiving a kidney transplant?

Did your mental health difficulty impact this experience?

Was there anything that made the process of receiving a kidney transplant more difficult?

Was there anything that made the process of receiving a kidney transplant easier?

What supports would be/would have been helpful at this stage?

**HCPs’ treatment of mental health difficulties**

Do your treating kidney healthcare providers know about your mental health difficulty?

Have you discussed your mental health difficulty with any member of the renal team? If no, do you mind me asking why that is?

If yes, do they have a good understanding of your mental health difficulty?

Have the renal team ever provided you with additional supports or services?

Has anyone discussed how your mental health difficulty might affect your kidney treatment? Or vice versa?

Have you ever been referred for psychological or psychiatric support through your physical health providers? Was this helpful/would it have been helpful?

**Social support**

Could you tell me about the social support you have? (friends/family/carer etc.)

**Mental health care services**

Are you involved with mental health services currently?

Were you involved with mental health care services when you were first diagnosed with kidney disease?

Is there any contact between mental health services and your kidney healthcare team?

In your experience, do mental health care professionals have a good understanding of kidney disease?

**Additional prompts if not already discussed:**

Do you think kidney disease patients who also have a mental health difficulty require more care than other kidney disease patients? If so, what kind of care?

Please share any ideas of how you think that we could improve the care of kidney patients with mental health difficulties.

What supports were most helpful for you? What supports would have been helpful for you?

What was least helpful to you?

In closing, is there anything else that you’d like to add that is relevant to the care of kidney disease patients with mental health difficulties? Anything that we didn’t ask?

Thank you again for your time and participation. (Stop recording)

**Family member interview schedule:**

**Background**

Age?

Gender?

Employment status?

Living situation?

Relationship status?

Ethnicity?

Could you tell me when your family member was diagnosed with kidney disease?

Have you been on dialysis?

Have you received a kidney transplant? When?

Do you have any other medical conditions?

what type of mental health difficulty do they have?

When diagnosed?

Have they been hospitalized?

Currently taking medication?

**Access to care**

Have they ever had difficulty accessing quality healthcare for your kidney disease?

Has your mental health difficulty made any aspect of kidney care more difficult?

*ESKD Diagnosis:*

Can you tell me a little bit about their diagnosis of kidney disease? When, by who, etc

What was the most difficult part of receiving their diagnosis?

Do you think their mental health difficulty made any aspect of diagnosis more difficult?

What helped care go well at this stage?

How could care be improved at this stage?

*For patients who have been/were on dialysis:*

How do/did they find dialysis? What is the most difficult aspect of attending dialysis for them?

Did you feel their mental health difficulty impacts dialysis?

In your experience, what helps dialysis go well for them?

What supports would be most helpful, to help dialysis go well for them?

How do/did they find the dietary and fluid restrictions? What is the most difficult aspect for them?

What makes sticking to diet and fluid restrictions easier? What supports would help them stick to diet and fluid restrictions?

*Transplant:*

Could you tell me a little bit about their experience of receiving a kidney transplant?

Did their mental health difficulty impact this experience?

Was there anything that made the process of receiving a kidney transplant more difficult?

Was there anything that made the process of receiving a kidney transplant easier?

What supports would be/would have been helpful at this stage?

**HCPs’ treatment of mental health difficulties**

Do their treating kidney healthcare providers know about your mental health difficulty?

Have you discussed their mental health difficulty with any member of the renal team? If no, do you mind me asking why that is?

If yes, do they have a good understanding of your mental health difficulty?

Have the renal team ever provided your family member with additional supports or services?

Has anyone discussed how their mental health difficulty might affect your kidney treatment? Or vice versa?

Have they ever been referred for psychological or psychiatric support through your physical health providers? Was this helpful/would it have been helpful?

**Social support**

Could you tell me about the social support they have? (friends/family/carer etc.)

Do you help your family with their kidney disease? If yes, how?

What supports would be most helpful for you?

**Mental health care services**

Are they involved with mental health services currently?

Were they involved with mental health care services when you were first diagnosed with kidney disease?

Is there any contact between mental health services and their kidney healthcare team?

In your experience, do mental health care professionals have a good understanding of kidney disease?

**Additional prompts if not already discussed:**

Do you think kidney disease patients who also have a mental health difficulty require more care than other kidney disease patients? If so, what kind of care?

Please share any ideas of how you think that we could improve the care of kidney patients with mental health difficulties.

What supports were most helpful for them? What supports would have been helpful for them?

What was least helpful?

In closing, is there anything else that you’d like to add that is relevant to the care of kidney disease patients with mental health difficulties? Anything that we didn’t ask?

Thank you again for your time and participation. (Stop recording)
